# Stand structure mediates the process of nutrient resorption in Chinese fir plantations during different stand developments

**DOI:** 10.3389/fpls.2025.1621379

**Published:** 2025-11-28

**Authors:** Yuanli Ouyang, Minxuan Chen, Qinxiu Huang, Fusheng Chen, Cancan Zhang, Wensheng Bu

**Affiliations:** 1 Key Laboratory of State Forestry Administration on Forest Ecosystem Protection and Restoration of Poyang Lake Watershed, Jiangxi Provincial Key Laboratory of Conservation Biology, College of Forestry, Jiangxi Agricultural University, Nanchang, China; 2 Jiulianshan National Observation and Research Station of Chinese Forest Ecosystem, Jiangxi Agricultural University, Nanchang, China

**Keywords:** development stage, stand structure, N and P resorption efficiency, nutrient utilization strategy, Chinese fir

## Abstract

**Introduction:**

Nutrient resorption refers to the process of transferring nutrients from senescing organs to living organs within plants for reuse. It is a key strategy enabling plants to conserve nutrients essential for growth and development. However, how stand structure influences nutrient resorption in different organs across stand developmental stages remains unclear.

**Methods:**

In this study, we conducted field investigations in 39 plots (20 m × 20 m) within Chinese fir plantations spanning four developmental stages, measuring nitrogen and phosphorus resorption efficiencies (NRE and PRE) in fine roots, twigs, leaves. We additionally calculated crown ratios, dead twig biomass, and relative growth rates within each plot.

**Results:**

Our results showed that stand density and relative growth rate decreased, while crown ratios increased and dead twig biomass initially increased rapidly before gradually declining during stand development. With stand aging, nitrogen concentrations increased in all organs, whereas phosphorus concentrations varied in aboveground vs. underground organs. Leaf NRE (NRE_L_) and absorptive/transportive root NRE (NRE_AR_ and NRE_TR_) peaked in young forests and progressively declined with stand development. Conversely, PRE in leaves and twigs (PRE_L_ and PRE_T_) reached maximum values in intermediate and mature forests. Trait network analysis revealed developmental stage-dependent shifts in central hub traits from NRE_AR_ to PRE_L_ and NRE_TR_, which reflected the change of nutrient demand during stand development. These findings demonstrate aboveground-belowground synergies in nutrient resorption, with fine roots mediating nutrient acquisition to support twig ans, accounting for the mass loss correctiond leaf growth. Developmental stages exerted dual effects: positively influencing stand structure while negatively impacting organ-level nutrient resorption. Stand development and structure similarly affected aboveground resorption efficiency (RE_AG_), whereas belowground resorption efficiency (RE_UG_) was mainly regulated by developmental stage. Stand structure positively influenced RE_AG_ and RE_UG_ through crown ratio suppression and stand density/relative growth rate/dead twig biomass enhancement.

**Discussion:**

Our results suggest management strategies for Chinese fir plantations: phosphorus addition in mature stands versus nitrogen supplementation in other stages, coupled with either increased initial planting density in young forests or preemptive understory canopy pruning to optimize nutrient resorption - particularly in nutrient-limited environments.

## Graphical Abstract

**Figure:**
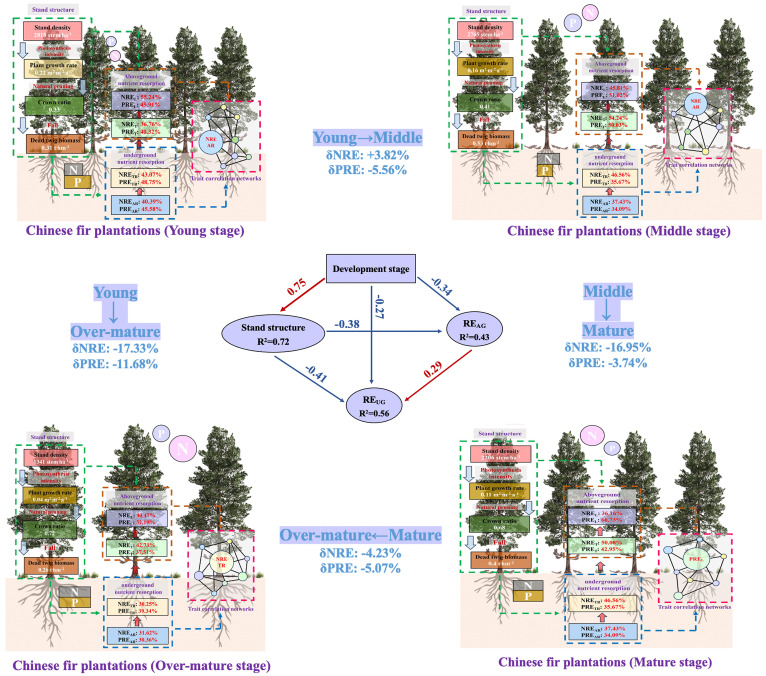
Schematic diagram suggesting the changes of N and P resorption efficiency with stand development and its driving factors on the aboveground and underground organs in Chinese fir plantations.

## 1 Introduction

In terrestrial ecosystems, nitrogen (N) and phosphorus (P) are the key limiting elements that influence plant growth, development, and nutrient cycling (Jiang et al., 2019). N cycling is a fundamental material cycling process within the biosphere, and plants primarily absorb nitrate or ammonium ions from the soil through their root systems in order to acquire N (Zhou et al., 2016). P cycling mainly occurs within soil, plants, and microorganisms and involves the absorption of available P from the soil by plants and the return of P from plant and animal residues to the soil for recycling (Zhao et al., 2011). Nutrient resorption is the transfer of nutrients from senesced plant tissues and reusing them for the creation of new living tissue (Wang et al., 2014). It not only improves nutrient use efficiency but also strengthens the adaptability of plants to nutrient-poor environments (Brant and Chen, 2015). For example, one study found that approximately 58.98% and 60.21% of N and P, respectively, are absorbed and used by plants through nutrient resorption (Jiang et al., 2019). In recent years, the process of nutrient resorption may change with stand development, as many physiological and ecological plant characteristics are related to stand development (Zhao et al., 2011). Previous studies have shown that with an increase in plantation age, litter decomposition rates slowed, and the amount of nutrient return decreased, thus reducing nutrient resorption efficiency (Brant and Chen, 2015). However, some previous studies have found that the nutrient resorption efficiency of twigs and leaves increases with increasing stand age, which is mainly controlled by the difference in nutrient concentrations before and after twig and leaf litter (Peri et al., 2006). The mechanism of nutrient resorption is extremely complicated because it involves many aspects and has a vital impact on the nutrient allocation patterns of almost all woody plants (Turner and Lambert, 2008). Knowledge of the dynamic pattern of nutrient resorption with developmental stage can help understanding whether plantations meet their nutrient reserves in the process of growth and maintain the normal nutrient allocation pattern.

Nutrient resorption is not only an important part of nutrient cycling, but also a key indicator of plant nutrient allocation (Zhou et al., 2016). Previous studies have shown that nutrient transfer, transport, and storage occur in different plant organs, among which leaves make the greatest contribution (Zhang et al., 2018). Leaf nutrient resorption efficiency decreases with increasing stand age because of the decrease in plant productivity and nutrient turnover rate, resulting in the storage of a large amount of nutrients in the leaf litter (Peri et al., 2006). However, some past studies have suggested that leaf nutrient resorption increases with stand development, because the nutrient concentration in mature leaves may decrease with increasing stand age, plantations reuse nutrients in the leaves through higher nutrient resorption efficiency (Zhang et al., 2018). However, it is related to nutrient limitation at different developmental stages. In the young stage, plantations are vulnerable to N limitation and have a high resorption efficiency of N, whereas in the mature stage, plantations are limited by P; therefore, the PRE is higher in this stage (Li et al., 2010). In summary, the mechanism by which leaf nutrient resorption changes with developmental stage remains unclear. Although previous studies have mainly focused on leaf nutrient resorption, considerable amounts of nutrients can also be resorbed through fine roots, twigs, and other organs (Yuan and Chen, 2012). Some studies have indicated that twig nutrient resorption in Chinese fir increases with stand development (Wang et al., 2014). Because twigs below the canopy usually transfer nutrients to other plant tissues by natural pruning, thereby improving nutrient resorption efficiency. Fine roots are important underground organs in plants that have fast turnover rate and high nutrient concentrations (Ren et al., 2023). With increasing stand age, fine root N resorption efficiency decreased, but P was not resorbed (Freschet et al., 2010). Other studies have found that fine root nutrient resorption efficiency increases with stand development (Yuan and Chen, 2012). This may be due to the significant increase in N:P and C:P in alive and dead roots, making fine roots more likely to be limited by P. To effectively protect P in the fine roots, plantations enhance P resorption efficiency. At present, there is no consensus on the dynamic patterns of nutrient resorption from different organs that change with an increase in stand age, which may differ due to the environment, tree species characteristics, and other factors (Liu et al., 2024).

In forest ecosystems, plant nutrient acquisition strategies cannot be underestimated, because plants usually do not have sufficient available nutrients to support their growth and reproduction, especially in nutrient-poor environments (Tully et al., 2013). Extensive studies have demonstrated that plants may adopt the strategy of “conservative consumption” (high nutrient resorption efficiency and low nutrient proficiency) in the early stage of growth, while “resource consumption” (a low nutrient resorption efficiency and high nutrient proficiency) is the key strategy in the late stage (Yan et al., 2006). However, the response of the nutrient resorption efficiency of plantations to stand age is not consistent, with positive, negative, or no significant effects, which may be related to tree species and nutrient status (Zeng et al., 2017). In recent decades, multiple plant strategies for above- and under-ground nutrient acquisition have been proposed, including leaf nutrient resorption (Hayes et al., 2014) and root nutrient acquisition (Hong et al., 2018). Previous studies have shown that there is a trade-off relationship between root nutrient acquisition and leaf nutrient resorption (Kou et al., 2017), and they have emphasized that the allocation of root and leaf nutrient resources depends on soil nutrient availability (Wright and Westoby, 2003). For example, plants prefer root acquisition strategies in nutrient-rich environments, because the cost of using nutrients in these environments is relatively low, whereas the opposite is true in nutrient-poor environments (Song et al., 2022). In addition, plants adapt to various nutrient limitations through strategies, which greatly contribute to forest ecosystems (Deng et al., 2019). A previous study found that when a tropical forest changed from N limitation to P limitation under the action of N deposition, plant P resorption efficiency was higher than the N resorption efficiency to alleviate the intensity of phosphorus limitation (Peng et al., 2023). Therefore, in a P limitation ecosystem, plants maintain their P supply and stoichiometric balance by coupling aboveground and underground nutrient acquisition strategies (Turner et al., 2018). Overall, it is vital to understand how plants balance aboveground nutrient conservation (leaf nutrient resorption) and underground nutrient acquisition (root nutrient capture) in order to alleviate nutrient limitations during stand development.

Stand structure determines the forest ecological function, and the law of stand structure provides a theoretical basis for forest management (Harrington et al., 2009). Stand density is not only a key factor that affects the composition, structure, and function of forest ecosystems (Russell et al., 2018), also affects the nutrient cycle of forest ecosystems (Dybzinski et al., 2013). For example, P resorption efficiency decreased and was negatively correlated with soil P content, indicating that P was the main nutrient-limiting element in plantations on the Loess Plateau (Xu et al., 2020). The growth rate hypothesis states that plant nutrient demand is closely related to the relative growth rate (Li et al., 2018). Fast-growing plants have a higher demand for nutrients, they usually improve nutrient resorption efficiency, indicating a positive correlation between plant relative growth rate and nutrient resorption (Achat et al., 2018). However, several studies have found a negative association between relative growth rate and P resorption efficiency (Li et al., 2013). These contradictory results highlight the need to study the effects of stand structure on nutrient resorption at different developmental stages. Litterfall is a nutrient and energy source for forest ecosystems (Wright and Westoby, 2003). Generally, the amount of persistent litter gradually increased with stand development, but some studies have suggested that the amount of litter increased first and then decreased with stand age (Zhou et al., 2015). In addition, the amount of litter and its nutrient concentration gradually increase with developmental stage (Selvaraj et al., 2017). An increase in stand age influences the amount of litter, a limited number of studies have indicated that increased litter input provides additional organic substrates for soil microorganisms, enhancing microbial activity and accelerating the release of N, P, and other nutrients, thereby indirectly supporting plant nutrient resorption (Jiang et al., 2023). However, excessive litter accumulation may form a thick layer that impedes oxygen diffusion and water infiltration, suppressing microbial decomposition activity and delaying nutrient release (Wang et al., 2025).

Over the past decades, to increase timber production, continuous cropping and other forest management measures have caused a significant decline in productivity of Chinese fir plantations (Jansons et al., 2013). Nutrient resorption has been shown to play a key role in improving stand productivity and enhancing nutrient acquisition (Zhou et al., 2016). During stand development, the shift in nutrient limitations is likely primarily driven by changes in plant nutrient demand and acquisition strategies, resulting in multiple potential transition pathways (Zhao et al., 2011). In this study, we have measured the changes in stand structure, nutrient concentration, and resorption efficiency across aboveground and underground organs during the developmental stages of subtropical Chinese fir plantations and explored the effects of stand development and stand structure on aboveground and underground nutrient resorption efficiency. We proposed the following three hypotheses: (1) In the young stage of stand growth, Chinese fir with a fast growth rate has a large demand for N; therefore, N resorption efficiency is high and gradually decreases in the mature stage. The demand for P in Chinese fir was mainly reflected in the flowering and fruiting stages, so the P resorption efficiency was high in the middle and mature stages, while the other stages were low; (2) because the roots absorbed N and P for the growth and utilization of twigs and leaves, the N and P resorptions of leaves and twigs theoretically had a synergistic effect with the roots; and (3) in theory, the developmental stage had a greater impact on nutrient resorption efficiency than the stand structure, because the change in stand structure was mainly caused by the process of development. The resolution of these issues will contribute to nutrient management strategies in the context of the degradation of Chinese fir plantations and provide novel insights for forest ecosystem management.

## 2 Materials and methods

### 2.1 Study site description and experimental design

The Chinese fir plantations investigated in this study were located at Guanshan National Forest Farm (27°13′-24°27′N, 115°45′-115°55′E), Yongfeng County, Jiangxi Province, China. The altitude ranges from 100 to 150 m above sea level. This site is characterized by a subtropical monsoon climate with mean annual temperature of 18°C, and mean annual precipitation of 1627 mm. The rainy season is mostly from April to June, whereas the dry season mainly occurs from July to October. The frost-free period is 279 days, with clear-sky duration per year of 1762 h and solar radiation of 4306 MJ·m^-2^. The soil type is classified as highly weathered Ultisol (Quaternary red clay soil), developed on slate, shale, and sandstone parent materials (Wang et al., 2019).

Since the 1970s, plantations of mainly *Cunninghamia lanceolata* and *Pinus elliottii* have been established in this area to meet the demand for timber for economic development. The Chinese fir plantation in this study was formed by hole digging and planting seedlings after manually removing all shrubs and herbs from the surface. The initial planting density was 3000 trees per hectare. Trees, shrubs, and grass that hindered tree growth tended to be removed during the first three years. Since then, no human activity has been conducted in order to allow the trees to grow and develop naturally. In July 2019, we designed an experiment with 3 replicates of 13 age classes with 4 development stages (young forests, middle forests, mature forests and over-mature forests) in a Chinese fir plantations with similar site conditions to clarify the differences of internal nutrient resorption in Chinese fir plantations among different development stages. Specifically, the age classes of Chinese fir plantations were divided according to fast-growing and high-yield timber plantation standards. Initially, it was set from 5- to 19-year-old in accordance with the age gradient of 2-year intervals, and then from 25- to 45-year-old following the age gradient of 5-year intervals: 5-, 7- and 9-year-old plantations as young forests; 11-, 13- and 15-year-old plantations as middle forests; 17-, 19- and 25-year-old plantations as mature forests, 30-, 35-, 40- and 45-year-old plantations as over-mature forests. Overall, 39–20 m × 20 m plots were isolated from each other through buffer zones of more than 100 m, and these plots had roughly the same slope. All plots at different developmental stages were investigated. First, the DBH, tree height, and crown diameter of all Chinese fir trees in the plot were measured, and then the stand density was calculated according to the plot area and the number of Chinese fir trees. Soil samples were collected from each plot according to the five-point method and brought back to the laboratory to obtain stand characteristics and soil properties (**Table 1**).

**Table 1:** Stand characteristics and soil physicochemical properties of Chinese fir plantations at different development stages. Mean ± standard error. The small letters indicate the differences among the four development stages (*p* < 0.05).

Variables	Stage			
	Young	Middle	Mature	Over-mature
Stand DBH (cm)	5.93 ± 0.14d	8.73 ± 0.17c	12.80 ± 0.13bb	19.47 ± 0.13a
Tree height (m)	5.51 ± 0.07d	8.30 ± 0.06c	10.71 ± 0.08b	13.26 ± 0.09a
Crown length (m)	1.55 ± 0.25d	3.39 ± 0.65c	7.31 ± 0.15b	9.61 ± 0.13a
Soil pH	4.63 ± 0.37a	4.60 ± 0.59a	4.52 ± 0.47b	4.62 ± 0.45a
SOC (g·kg^-1^)	18.16 ± 0.36b	16.89 ± 0.3c	17.75 ± 0.5bc	22.39 ± 0.31a
Total N (g·kg^-1^)	1.54 ± 0.14c	1.74 ± 0.06ab	1.65 ± 0.03b	1.81 ± 0.02a
Total P (g·kg^-1^)	0.78 ± 0.04b	0.77 ± 0.04b	1.05 ± 0.2a	0.74 ± 0.03b
NH_4_^+^-N (mg·kg^-1^)	6.82 ± 0.22c	7.78 ± 0.32b	9.35 ± 0.21a	7.05 ± 0.19c
NO_3_^–^N (mg·kg^-1^)	2.66 ± 0.06c	3.43 ± 0.03b	4.04 ± 0.06a	3.58 ± 0.05b
Available P (mg·kg^-1^)	3.95 ± 0.08a	3.17 ± 0.06b	3.14 ± 0.08b	3.17 ± 0.07b
Mineral N/Available P	2.40 ± 0.06c	3.59 ± 0.56b	4.27 ± 0.28a	3.35 ± 0.18b

### 2.2 Sample collection

According to the results of the plot inventory, five Chinese fir trees from each plot located near the four corners and center points, with a diameter close to the average breast height of the plot, were used to collect samples of leaves, twigs, canopy litterfall, rhizosphere soil, and fine roots. Fresh twigs with live leaves were collected from the upper two-thirds of the tree canopies in multiple directions, and canopy litterfall was sampled using a 10 meter-tree trimmer. All dead twigs and leaves in the canopy were collected upward from the position of the lowest dead branches on the trunk. If the positions of fresh twigs and litterfall were too high, professional tree climbers were hired to climb trees using tree-climbing foot buckles and collect dead branches and fresh twigs with alive leaves. The fresh weights of alive twigs, leaves, dead twigs, and leaves were measured in the field using a portable electronic balance. To obtain an accurate number of dead twigs and leaves, a portion of the collecting dead branches and leaves was selected and brought back to the laboratory. Dead twigs were taken from the upper, middle, and lower three branches of the collection position to measure fresh weight and dry weight after drying. Dead leaves (500 g) were taken from each of the extracted twigs, weighed approximately 1500 g fresh weight, and brought back for drying. The water content of dead twigs and leaves of each Chinese fir was calculated, and the number of dead twigs and leaves was calculated by combining the fresh weights of dead twigs and leaves obtained in the field. Subsequently, the twigs and leaves of Chinese fir were graded, second-order twigs and leaves were selected as the live twigs and leaves, the primary rationale for selection stems from its advanced leaf age (>1 year), classifying it as an old leaf, thereby making it more suitable for our study objects (Chen et al., 2015). All of the samples of alive and dead twigs and leaves were oven-dried to constant weight (65°C for over 72 h). After grinding, samples of different organs were obtained through a 0.15 mm sieve.

In addition, we sampled four soil blocks (30 cm in length, 30 cm in width, and 20 cm in depth; 20 blocks were collected in each plot) around the canopies of each target tree 50 cm from the tree trunk and carefully obtained intact fine roots (<2 mm in diameter). We obtained rhizosphere soil that adhered to the surface of fine roots within 4 mm of the rhizosphere after handshaking (Phillips and Fahey, 2008). The rhizosphere soil samples of each plot were mixed into a composite sample and immediately transported to the laboratory to determine available nutrients (NH_4_^+^-N, NO_3_^−^-N and available P). Alive and dead fine roots can be distinguished based on their shape, elasticity, and color. Live fine roots are light brown with good elasticity, whereas dead fine roots are dark brown and also easy to pull (Ren et al., 2023). Subsequently, the fine roots were further divided into absorptive roots (AR: 1–3 order root) and transportive roots (TR: 4 order roots or above) (Pregitzer et al., 2002). Alive and dead absorptive roots and transportive roots in each soil block were washed with ultrapure water for scanning and drying. Finally, the absorptive roots and transportive roots of 20 soil blocks in each plot were combined separately into composite samples and ground to pass through a 0.15 mm sieve for chemical analysis.

### 2.3 Chemical analysis

The pH was determined using a potentiometric method, and the sample was oscillated in a 1:5 soil: aqueous solution for 0.5 hours. Soil organic carbon (SOC) was measured using the potassium dichromate (K_2_Cr_2_O_7_) electricity plate heating method. Soil ammonium nitrogen (NH_4_^+^-N) and nitrate nitrogen (NO_3_^–^N) were extracted with a 2 mol.L^-1^ KCl solution at a soil-to-solution ratio of 1:5 (Chen et al., 2015). Soil available phosphorus (AP) was extracted with 0.03 mol.L^-1^ NH_4_F plus 0.025 mol.L^-1^ HCl solution, the ratio of soil to solution was 1:10, and the available nutrients were detected using a continuous flowing analyzer. Total nitrogen (TN) and total phosphorus (TP) were analyzed using the Kjeldahl and molybdenum-antimony colorimetric methods, respectively, after the samples were digested with H_2_O_2_-H_2_SO_4_.

### 2.4 Calculation of relevant parameters

The crown ratio (CR) was used as an index of natural pruning intensity (Fu et al., 2017):

(1) CR= CL/TH

Where CR represents the crown ratio; the higher the index, the lower the natural pruning intensity; CL represents the crown length; and TH represents the tree height.

Relative growth rate (RGR) (Li et al., 2018), and dead twig biomass (Zhou et al., 2021) were calculated as follows:

(2) $$\text{RGR }\left({\text{m}}^{2}\cdot {\text{m}}^{-2}\cdot {\text{a}}^{-1}\right)=\text{ ln }\left({\text{DBH}}_{2}\right)-\text{ln }\left({\text{DBH}}_{1}\right)/\left({\text{t}}_{2}-{\text{t}}_{1}\right)$$

(3) $${\text{W}}_{i\text{ dead twig }(i=1,2\ldots \ldots ,\text{n)}}={\text{FW}}_{i\text{ dead twig}}\times (1-{\text{MC}}_{i\text{ dead twig}})$$

(4) $${\text{W}}_{\text{ dead twig}}\;(\text{t}\cdot {\text{hm}}^{-2})\;=\;\sum {\text{W}}_{i\text{ dead twig (}i=1,2\ldots \ldots ,\text{n)}}/\text{A}$$

Where DBH_1_ and DBH_2_ represent the average diameters of all of the trees in each plot at times t_1_ and t_2_, respectively. W*i*_dead twig_ is the dead twig biomass of the *i*th tree (*i* = 1,2,…n) in the plot, FW is the fresh weight of the dead twig of the *i*th tree, MC is the moisture content of the dead twig of the *i*th tree, W_dead twig_ is the total dead twig biomass of all individuals in the plot, and A is the plot area.

Nutrient resorption efficiency (RE) is the percentage reduction in nutrient concentrations between live (Nu_alive_) and dead (Nu_dead_) organs, accounting for the mass loss correction factor (MLCF) (Sun et al., 2023):

(5) $$\text{RE }\left(\%\right)\;=\;\left(1-{\text{Nu}}_{\text{dead}/}{\text{Nu}}_{\text{alive}}\times \text{MLCF}\right)\times 100$$

where Nu_alive_ represents the nutrient concentration in alive twigs, leaves, absorptive roots, and transportive roots, and Nu_dead_ represents the nutrient concentration in the dead twigs, leaves, absorptive roots, and transportive roots. MLCF is a correction factor that accounts for mass loss during organ senescence.

### 2.5 Statistical analysis

All data were tested for homogeneity of variance prior to statistical analysis. One-way analysis of variance and least significant difference methods were used to analyze the differences in stand structure, nutrient concentration, and nutrient resorption efficiency among the four developmental stages. Trait networks are tools used to represent the relationships among plant traits. In these networks: nodes represent different plant traits (such as nutrient resorption efficiencies of aboveground and underground organs), and edges represent the correlations between these traits (Wei et al., 2023). Specifically, the Spearman correlation coefficients are employed to measure the strength of correlations among traits. Network centrality is an important index in network analysis, which is applied to evaluate the influence of a node (plant trait) in the whole networks (Wei et al., 2023). Betweenness centrality/centralization is a commonly used network centrality index measure that assesses the extent to which a node acts as a ‘bridge’ within trait network (Messier et al., 2017). It calculates the frequency at which a node appears on the shortest paths between all pairs of nodes. If a node lies on the shortest paths between many other nodes, its betweenness centrality is high, indicating that it plays a crucial connecting role in the trait network. In this study, we built trait networks to investigate the coordination of nutrient resorption between aboveground and underground organs across different developmental stages. The IGRAPH package was utilized to visually represent statistically significant relationships of nutrient resorption between aboveground and underground organs, and network centrality was computed for nutrient resorption between aboveground and underground organs and its trait network using the betweenness function in the IGRAPH R package (Wei et al., 2023). A hub trait refers to a trait in a network that possesses the highest betweenness centrality. This trait plays a pivotal bridging role within the network, coordinating variations among multiple traits, and it exerts a significant influence on the stability of the entire trait network (Messier et al., 2017). Path length is the number of edges in the shortest path between two nodes in the network. Betweenness centrality is closely related to path length because nodes with high betweenness centrality typically lie on many shortest paths, thereby influencing the overall structure and efficiency of trait network (Messier et al., 2017). To analyze the impact of stand development on the topological properties of trait network, we computed some topological properties (such as edge, path length, and betweenness centrality) utilizing IGRAPH R package in the R 4.1.3. Structural equation modeling (SEM) was performed to explore how stand structure responds to stand development and affects nutrient resorption efficiency (Eisenhauer et al., 2015). Considering the developmental stage, we quantified the young stage as 1, the middle stage as 2, the mature stage as 3, and the overmature stage as 4 in the SEM. The development stage was set as an exogenous variable, and RE_L_, RE_T_, RE_AR_ and RE_TR_ were considered endogenous variables. All predictors were standardized to have a mean of 0 and a standard deviation of 1 to improve the interpretability of the regression coefficients (Eisenhauer et al., 2015). Maximum likelihood estimation was used to test whether the data fit the model. A total standardized effect was applied to determine the relative importance of the developmental stage and also the stand structure on nutrient resorption efficiency and to identify the key drivers that affected internal nutrient cycling. The standard *p*<0.05 level was used throughout as the threshold for statistical significance. All statistical analyses were performed using SPSS version 26.0. Network and structural equation modeling were performed using R 4.1.3.

## 3 Results

### 3.1 Variations in stand structures with the process of developmental stages

**Figure 1:**
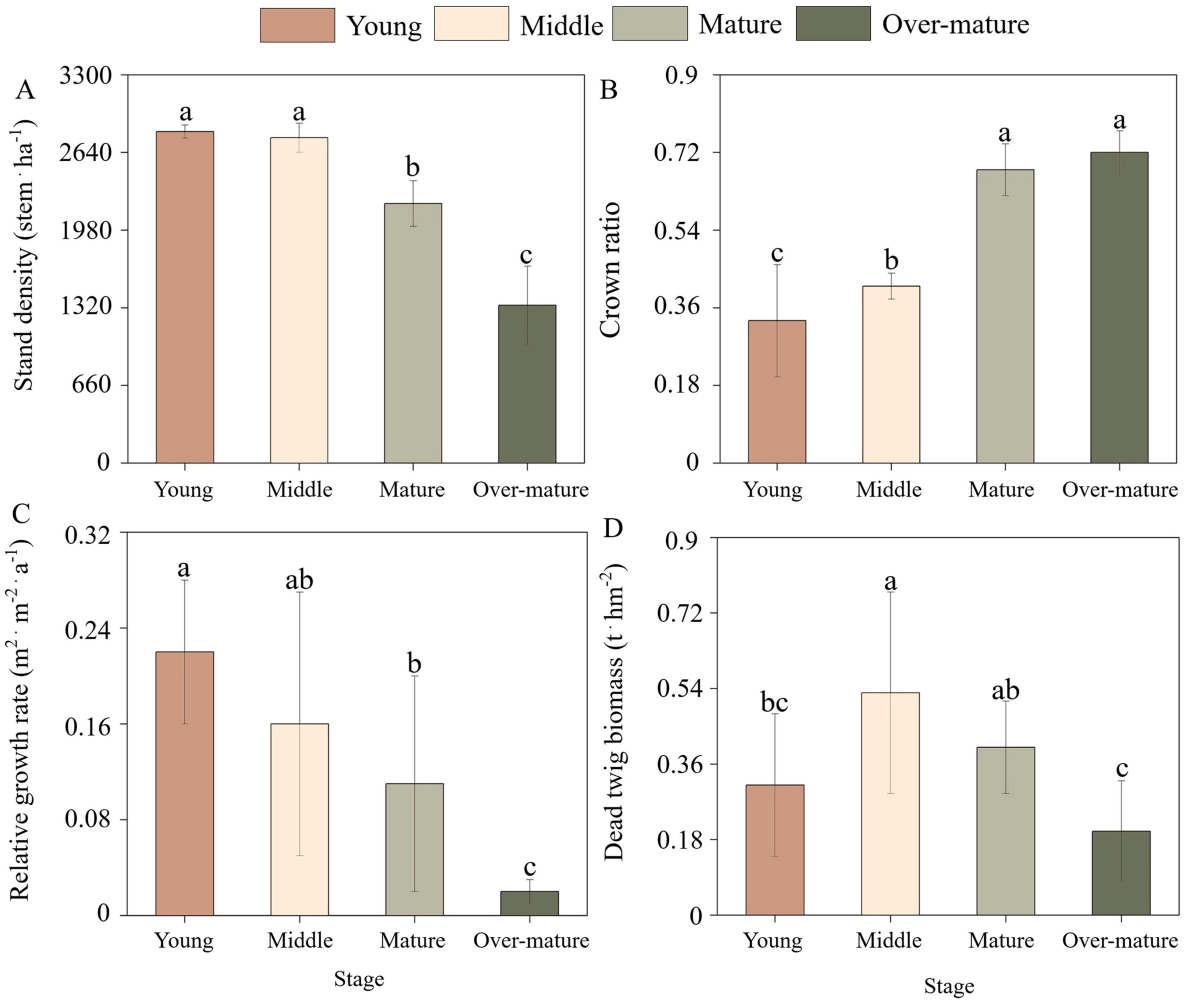
Stand structures of Chinese fir plantation across the different development stages. **(A)** Stand density with four (young, middle, mature, over-mature) development stages. **(B)** Crown ratio with four development stages. **(C)** Relative growth rate with four development stages. **(D)** Dead twig biomass with four development stages. The small letters indicate the differences among four development stages (*p* < 0.05).

The developmental stage was found to have significant effects on stand density, natural pruning intensity, relative growth rate, and dead twig biomass (**Figure 1**). The stand density decreased with the developmental stage, and the stand densities in the young, middle, mature, and over-mature forests were 2818, 2765, 2206 and 1341stem.ha^-1^, respectively. However, the crown ratio increased with the process of development stage (Equation 1). The relative growth rate decreased linearly with the process of development stage from 0.22 to 0.02 (Equation 2). The relative growth rate in the mature forest was significantly lower than that in the young forest and higher than that in the over-mature forest. The dead twig biomass in the middle forest was significantly higher than that in the young and over-mature forests but was not different from that in the mature forest (Equations 3, 4). In summary, stand density and relative growth rate decreased and natural pruning intensity increased during the stand development stage. Dead twig biomass increased rapidly and then decreased gradually during the stand development stage.

### 3.2 Variations in nutrient concentrations of alive and dead organs with the process of developmental stages

**Figure 2:**
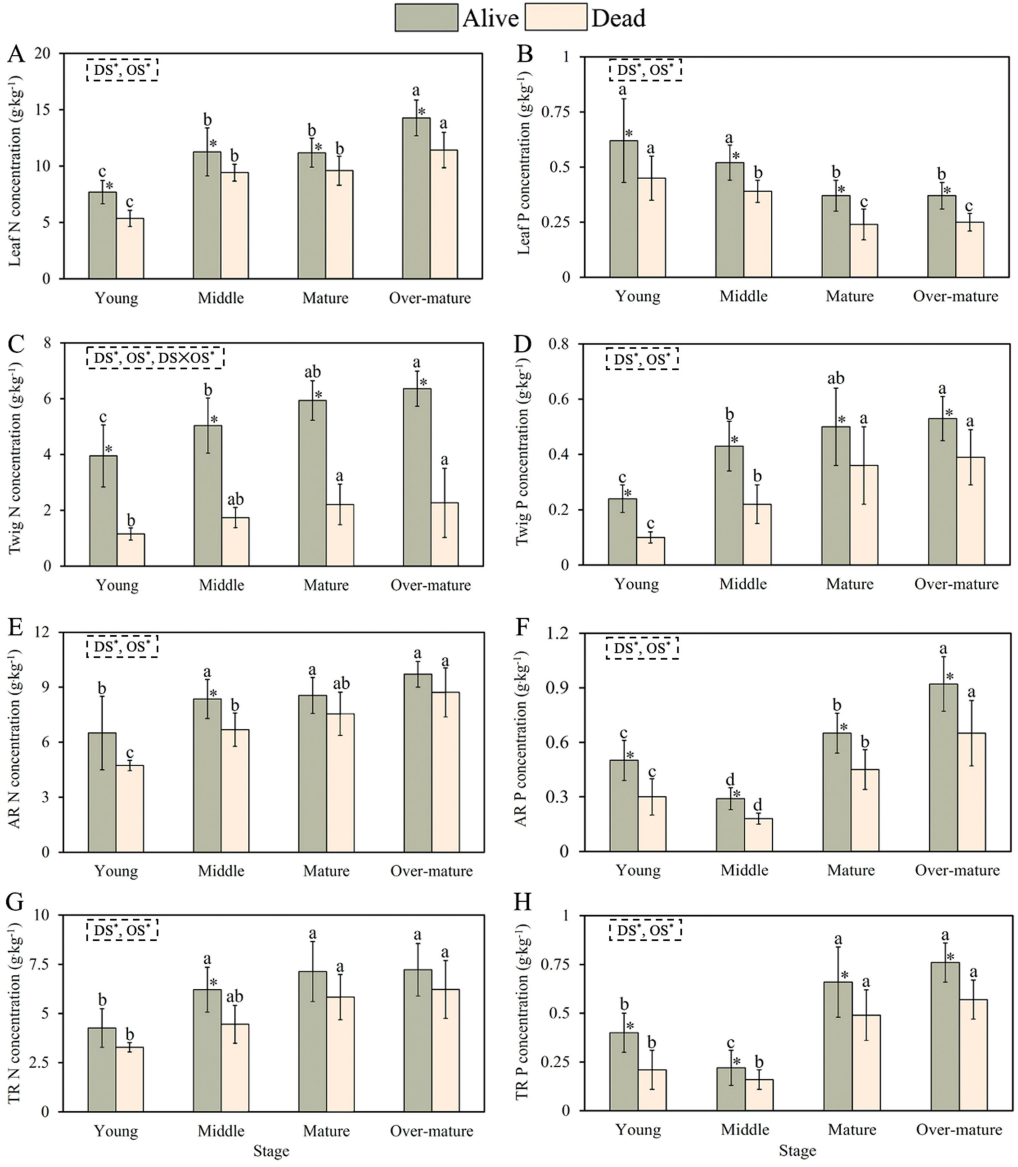
Nutrient concentrations of alive and dead leaves, twigs and fine roots across the different development stages. **(A)** Leaf N concentration with four development stages. **(B)** Leaf P concentration with four development stages. **(C)** Twig N concentration with four development stages. **(D)** Twig P concentration with four development stages. **(E)** AR N concentration with four development stages. **(F)** AR P concentration with four development stages. **(G)** TR N concentration with four development stages. **(H)** TR P concentration with four development stages. The small letters indicate the differences among the four development stages (*p* < 0.05). * represents the differences between the alive and dead organs within the same development stage. DS, development stage; OS, organ survival; L, leaf; T, twig; AR, absorptive root; TR, transportive root.

The N and P concentrations in the leaves, twigs, and absorptive and transportive roots were significantly influenced by the development stage (DS) and organ survival (OS) (**Figure 2**). The N concentration of alive and dead leaves increased, whereas the P concentration of alive and dead leaves decreased during the developmental stage (**Figures 2A, B**). The N and P concentrations in alive leaves were higher than those in dead leaves at all developmental stages. The N and P concentrations of the alive and dead twigs increased with developmental stage. Moreover, the stoichiometry of the alive twigs was significantly higher than that of the dead twigs at all developmental stages (**Figures 2C, D**).

N concentrations of alive, dead absorptive, and transportive roots increased with developmental stage, and N concentrations of the alive absorptive and transportive roots were significantly higher than those of the dead absorptive and transportive roots in the middle forest (**Figures 2E, G**). The P concentrations in alive, dead absorptive, and transportive roots first decreased and then increased with the developmental stage. The P concentrations in the alive absorptive and transportive roots were higher than those in the dead absorptive and transportive roots at all developmental stages (**Figures 2F, H**).

### 3.3 Variations in nutrient resorption efficiencies of plant organs with the process of developmental stages

**Figure 3:**
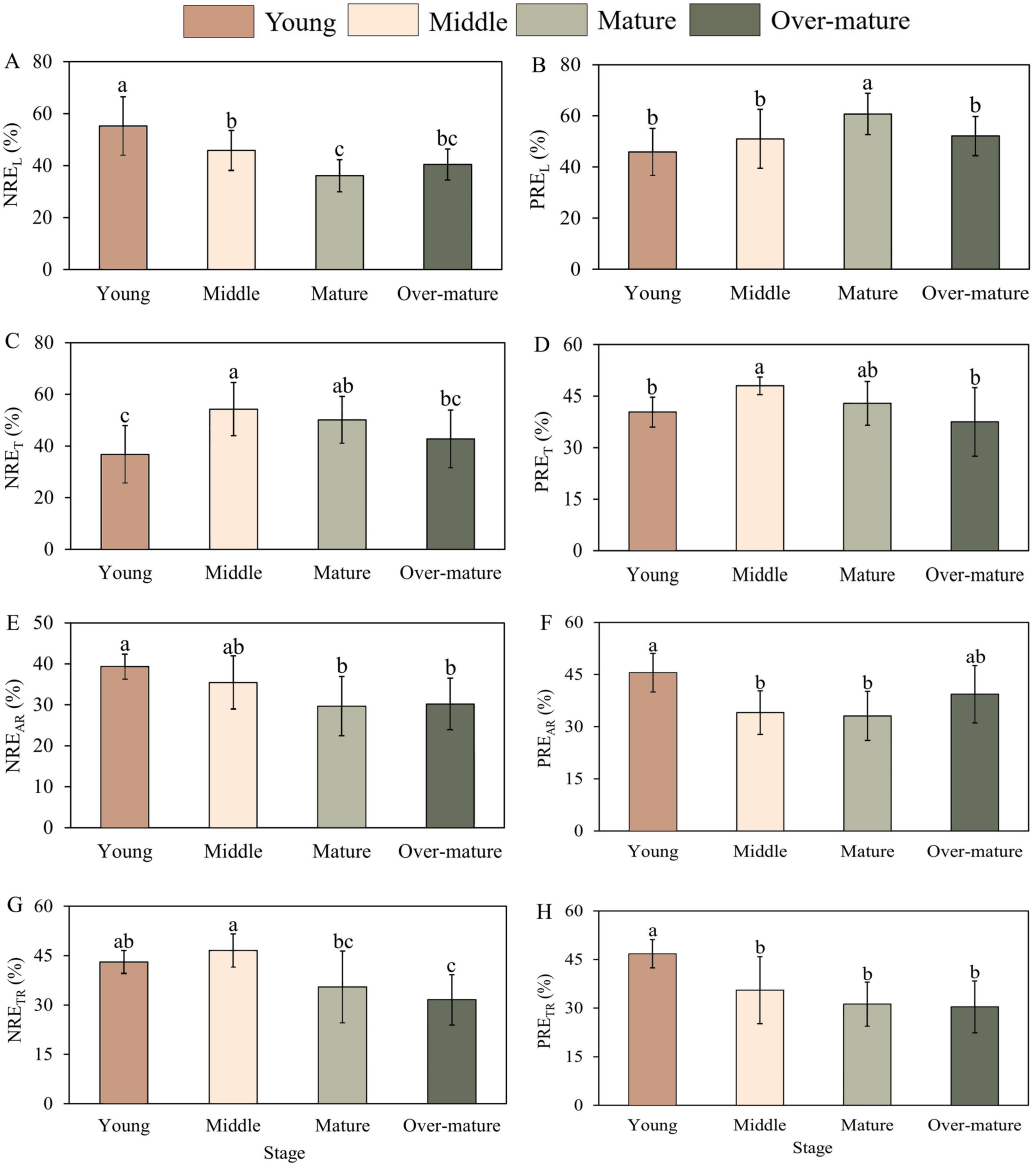
Nutrient resorption efficiencies of leaf, twig and fine roots across the different development stages. **(A)** NRE_L_ with four development stages. **(B)** PRE_L_ with four development stages. **(C)** NRE_T_ with four development stages. **(D)** PRE_T_ with four development stages. **(E)** NRE_AR_ with four development stages. **(F)** PRE_AR_ with four development stages. **(G)** NRE_TR_ with four development stages. **(H)** PRE_TR_ with four development stages. The small letters indicate the difference among the four development stages (*p*<0.05). RE_L_, RE_T_, RE_AR_ and RE_TR_ represent the resorption efficiencies of leaf, twig, absorptive root and transportive root, respectively.

There were significant differences in the aboveground and underground nutrient resorption efficiencies among the different developmental stages (**Figure 3**) (Equation 5). The NRE_L_ in the middle-aged forest was significantly lower than that in the young forest and higher than that in the mature forest. The PRE_L_ first increased and then decreased during the development stage from 45.91% to 60.73% (**Figures 3A, B**). The PRE_L_ in the mature forest was significantly higher than that in the other forests. NRE_T_ and PRE_T_ in the middle forest were the highest and were significantly higher than those in the young and over-mature forests (**Figures 3C, D**). The NRE_AR_ in the young forest was found to be significantly higher than that in the mature and over-mature forests, whereas the PRE_AR_ in the young forest was significantly higher than that in the middle and mature forests (**Figures 3E, F**). The NRE_TR_ first increased and then decreased with the developmental stage from 21.6% to 33.07% (**Figure 3G**). The NRE_TR_ in the middle forest was significantly higher than that in the mature and over-mature forests. The PRE_TR_ decreased with developmental stage and was significantly higher in the young forest than in the other forests (**Figure 3H**). Overall, the highest NRE_L_, NRE_AR_, PRE_AR_, and PRE_TR_ and the lowest PRE_L_ and NRE_T_ were observed in the young forest. The highest NRE_T_ and PRE_T_ NRE_TR_ in the middle-aged forest, whereas the highest PRE_L_ and lowest NRE_L_, NRE_AR_, PRE_AR_ in the mature forest. The lowest PRE_T_, NRE_TR_, and PRE_TR_ values were observed in the over-mature forest. NRE_L_, NRE_AR_, and PRE_TR_ declined with developmental stage; NRE_T_, PRE_T_, and NRE_TR_ increased from young forest to middle-aged forest and decreased from middle-aged forest to over-mature forest.

### 3.4 Differences in trait networks with the process of developmental stages

**Figure 4:**
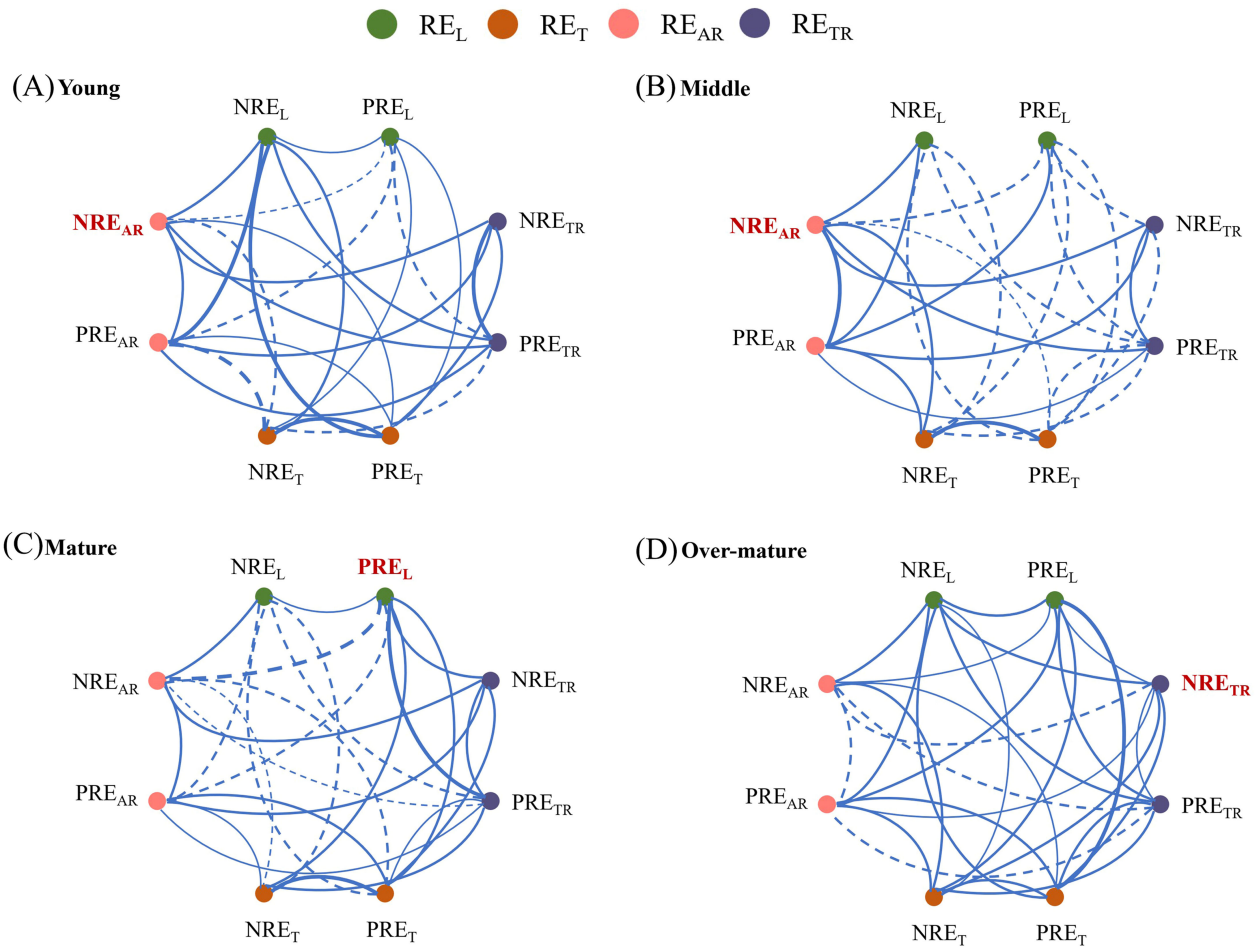
Trait networks among aboveground and underground nutrient resorption efficiency across different development stages. **(A)** Trait networks in young forest. **(B)** Trait networks in middle forest. **(C)** Trait networks in mature forest. **(D)** Trait networks in over-mature forest. Solid and dashed edges indicate positive and negative correlations, respectively. Only significant correlations are shown (*P* < 0.05), with the strength of the correlation being represented by edge thickness. The red bold in the networks show the highest Betweenness centrality (the number of shortest paths from all nodes to all other nodes through a focal node). The green, yellow, pink and purple circles denote leaf, twig, absorptive root and transportive root nutrient resorption efficiency, respectively. All nutrient resorption efficiency values were log10-transformed to improve the linearity of correlation among traits.

**Figure 5:**
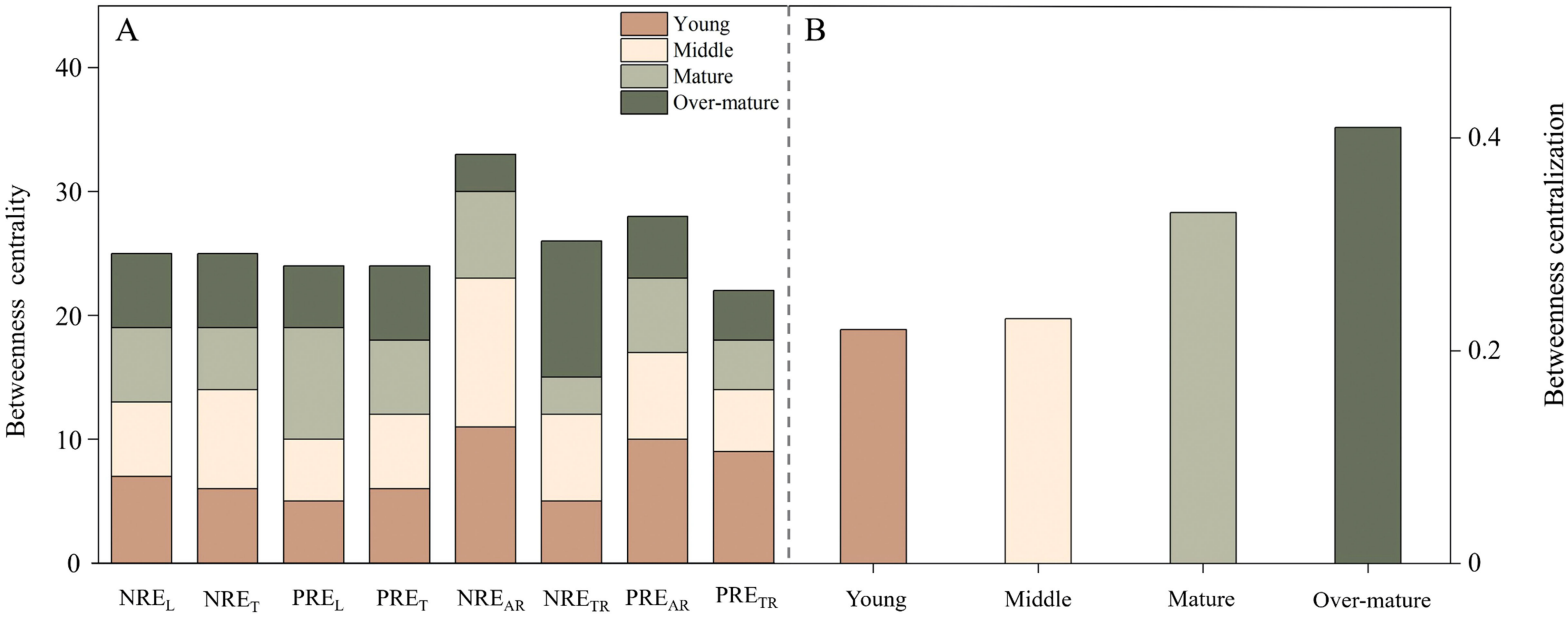
The Betweenness centrality **(a)** and centralization **(b)** of nodes response to aboveground and underground nutrient resorption efficiencies across different development stages.

The developmental stage did not change the overall connectedness of the trait network but altered the betweenness centrality (**Figure 4**). The effect on stand development on the proportion of connectivity among traits was slight, with 23 significant aboveground-underground resorption efficiency relationships in the middle forest and 25 in other forests. In response to the process of developmental stages, the central hub traits with the highest betweenness centrality moved from NRE_AR_ to PRE_L_ and NRE_TR_ (**Figure 5**). In other words, NRE_AR_ was the central hub trait in young and middle forests, but PRE_L_ and NRE_TR_ emerged as the central hub trait in mature and over-mature forests, respectively (**Figure 5A**). Moreover, the betweenness centralization increased with stand development (**Figure 5B**).

### 3.5 Drivers of nutrient resorption efficiency

**Figure 6:**
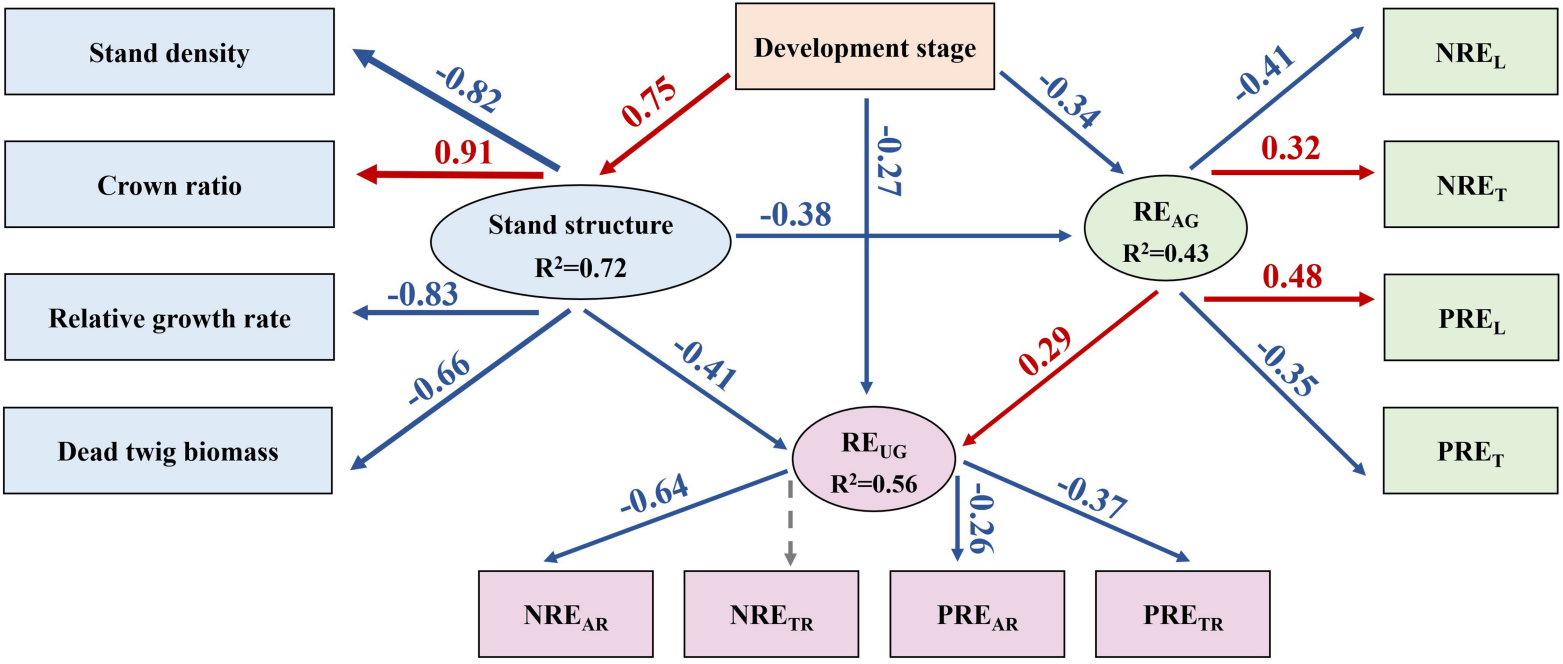
The structural equation models of the effects of the development stage and stand structure on the aboveground (AG) and underground (UG) nutrient resorption efficiency (*χ2* = 25.515; *df* = 24, *P* = 0.33; *CFI* = 0.962; *RMSEA* = 0.047; *AIC* = 92.972 in SEM) in Chinese fir plantations. Note: Black and gray arrows indicate significant positive and negative relationships, respectively. The arrow width denotes the strength of the causal influence and the numbers are standardized path coefficients. The solid arrows represent significant (*p*<0.05) and the dashed arrows represent non-significant (*p*>0.05) relationships.

The SEM was employed to assess the direct and indirect relationships between RE_UG_ and RE_AG_, development stage and stand structure. Overall, development stage played a direct and indirect negative role on RE_AG_ and RE_UG_, while stand structure positively and directly impacted RE_AG_ and RE_UG_. Notably, RE_AG_ and RE_UG_ exhibited significant positive correlation. In terms of the aboveground nutrient resorption (RE_AG_) process, the developmental stage directly and negatively affected the RE_AG_ and indirectly had a negative effect on the RE_AG_ through a positive effect on stand structure (**Figure 6**). For stand structure, the developmental stage indirectly had a positive effect on the crown ratio, which indirectly indicated that the natural pruning intensity decreased with increasing stand age. Moreover, the developmental stage indirectly had a negative effect on stand density, relative growth rate, and dead twig biomass. Moreover, the developmental stage indirectly had a negative effect on NRE_L_ and PRE_T_ and a positive effect on NRE_T_ and PRE_L._ In terms of the underground nutrient resorption (RE_UG_) process, the developmental stage directly and negatively affected RE_UG_, and not only indirectly had a negative effect on RE_UG_ through a positive effect on stand structure, but also indirectly had a positive effect on RE_UG_ through a negative effect on RE_AG_. For RE_UG_, development stage indirectly had a negative effect on NRE_AR_, PRE_AR_, and PRE_TR._

**Figure 7:**
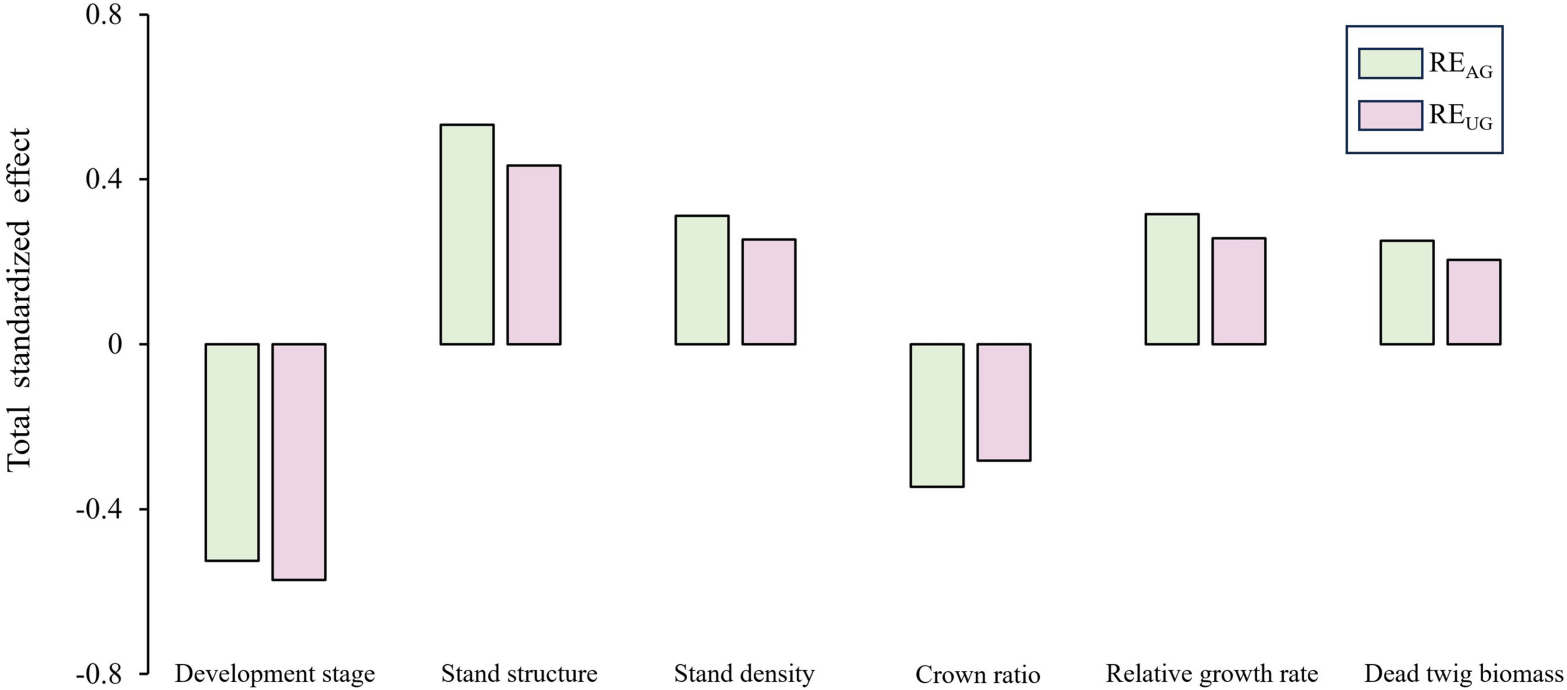
Total standardized effect of factors on the aboveground-underground nutrient resorption efficiency.

The results of the standardized total effects (including direct and indirect effects) indicated that developmental stage had a negative effect on RE_AG_ and RE_UG_ (**Figure 7**). Furthermore, the stand structure had a positive effect on RE_AG_ and RE_UG_ through the negative effect of crown ratio and the positive effects of stand density, relative growth rate, and dead twig biomass.

## 4 Discussion

### 4.1 Effects of stand development on stand structure and nutrient concentration

This study revealed that stand density and relative growth rate gradually decreased with increasing stand age, which may be closely associated with the growth characteristics of the tree species (Harrington et al., 2009). A higher number of individuals were present in the young forest, coupled with limited resources available within the stand, leading to intense competition among them, exhibiting faster growth rates (Di et al., 2018). The less dominant individuals gradually died during the developmental stage, stand growth became more stable after the mature forest, thereby gradually decreasing the growth rate (Li et al., 2022). Crown ratio was lowest in the young forest and gradually increased with increasing stand age, but dead twig biomass exhibited an initial increase, followed by a decrease with stand development, with the highest amount occurring in the middle forest. This was mainly related to the limitation of the crown extension space among the individuals in the stand and the change in twig mortality in the lower part of the crown (Shah et al., 2024). Specifically, because of the high stand density at the young stage, the mutual shading between the crowns of each individual tree leads to the gradual death of the lower branches of the crown, thus causing higher natural pruning intensity and more dead twigs in the canopy (Fu et al., 2017). Stand density gradually decreased with the process of stand development, which alleviated the spatial limitation of the canopy growth of the surviving individuals to a certain extent, finally decreasing the natural pruning intensity and the mortality of the dead twigs (Zhou et al., 2021).

Our results showed that the nutrient concentration in the living organs of Chinese fir trees was higher than that in dead organs. This could be due to the transfer of nutrients from the organ before its death to the living organs, which is an essential strategy for plants to conserve nutrients and maintain the internal nutrient balance, as well as an adaptive mechanism for coping with nutrient-poor environments (Milla et al., 2005). As the primary site of photosynthesis in plants, live leaves accumulate substantial amounts of starch, sugars, and organic compounds and have relatively high nutrient concentrations (Huang et al., 2007). In this study, the N concentration in the leaves, twigs and fine roots, including absorptive and transportive roots increased with the development of Chinese fir, which is consistent with previous studies (Peng et al., 2023). In the young forest, the conductive tissues and other structures are not fully developed, necessitating a strong capacity for cell division to synthesize abundant proteins, thereby increasing N concentration (Tang et al., 2013). Furthermore, during stand growth, cellular metabolism and photosynthesis continually increase tree biomass, enhancing their capacity to absorb nutrients, which in turn leads to a sustained increase in N concentration in leaves (Zhang et al., 2018).

The P concentration in leaves decreased with the developmental stage. During the young and middle stages, the rapid growth of Chinese fir requires more DNA, RNA, and ATP for photosynthesis, thereby consuming a substantial amount of P within the plant (Yan et al., 2018). The physiological functions of the Chinese fir tended to decline with increasing stand age. In mature and over-mature forests, P may be transferred to flowers and fruits, thus leading to a gradual decrease in P concentration in the leaves (Hayes et al., 2014). With the process of the developmental stage, more P is transferred from the leaves and stored in woody tissues (including stems, twigs, and roots) (Crous et al., 2019). In contrast, the P concentration in twigs gradually increased with stand development. Chinese fir has a substantial demand for nutrients and space because of its rapid growth, which leads to a sustained increase in its nutrient concentrations (Di et al., 2018). Changes in nutrient concentrations of twigs during stand development may be related to the dilution effect (Zhou et al., 2021). In addition, nutrient concentrations of alive twigs had been at a relatively high level with increase of stand age, and the change in nutrient resorption efficiency was relatively stable, so that nutrient concentrations of dead twigs was also high (Lü et al., 2012). In addition, the P concentration of fine roots initially decreased and then increased with stand development. To ensure rapid growth in young and middle forests, Chinese fir allocates more P to the construction and synthesis of aboveground photosynthetic organs, resulting in relatively lower P concentrations in fine roots (Yuan and Chen, 2012). With the process of the developmental stage, plant require substantial amounts P to increase branching and surface areas of fine roots, improving their water and nutrient acquisition capacity, thereby supporting rRNA synthesis and supplying energy for vital metabolic processes (Lambers, 2022).

### 4.2 Nutrient resorption efficiency and trait networks in response to stand development

NRE_L_ and NRE_AR_ were found to be high in the young forest and gradually decreased in the mature forest, which is consistent with the first hypothesis. First, some studies have found that nutrient limitation during different developmental stages of plants is a crucial factor that causes variations in nutrient resorption efficiency (Brant and Chen, 2015). In young forests, plantations are more prone to N limitation, resulting in a higher NRE (Zhao et al., 2011). Second, the nutrient concentration in living organs has an important effect on nutrient resorption efficiency (Zeng et al., 2017). In the young forest, N concentrations in live leaves and fine roots were low. To maintain normal growth and development, plants need to adjust their nutrient utilization strategies and reuse nutrients through nutrient resorption (Yan et al., 2006). During stand development, the nutrient concentrations of live leaves and fine roots gradually increased, and the NRE decreased. PRE_L_ and PRE_T_ were high in the middle and mature forests and lower in the other stages. In the young forest, Chinese fir trees grew at a faster rate, requiring more P to synthesize rRNA to meet its nutrient demands (Peng et al., 2023). In the mature forest, Chinese fir trees entered the flowering and fruiting stages, and the aboveground organs became an effective repository of P, which reduced the PRE.

In addition, NRE_T_ was highest in the middle forest and lowest in other stages, which was contrary to the first hypothesis. In the middle forest, Chinese fir trees were in a crucial stage for natural pruning, leading to a low nutrient concentration in twigs and a continuous increase in dead twig biomass, after which nutrient concentration accumulated in the Chinese fir plantations can maintain its normal growth (Yan et al., 2016). Therefore, NRE_T_ first increased and then decreased with stand development. PRE_AR_ and PRE_TR_ were the highest in the young forest and gradually decreased with increasing stand age. On the one hand, fine roots in the young forest typically generate greater specific root length and specific surface area, which increases their contact area with soil and enhances nutrient absorption efficiency (Freschet et al., 2010). On the other hand, plants exhibit high nutrient demand during initial growth phases, and fine roots demonstrate elevated metabolic activity, enabling rapid nutrient translocation to other plant parts (Li et al., 2013). Consequently, fine roots in the young forest prioritize nutrient acquisition, resulting in relatively high resorption efficiency. With the increase of stand age, the P stored in transportive roots could satisfy the needs of the flowering and fruiting stages, so it did not need to resorb more nutrients from other organs.

Stand development led to a change in the central hub traits of the network, moving from NRE_AR_ to PRE_L_ and NRE_TR_. This result reflects the increased nutrient demand during stand development (Liu et al., 2024), which might shift competitive nutrient acquisition from underground N in the young and middle forests to aboveground P in the mature forest, eventually transitioning to underground N storage in the over-mature forest. In both young and middle forests, NRE_AR_ demonstrated the highest betweenness centrality within the trait network, indicating that it plays a vital connecting role in the trait network of above developmental stages. Chinese fir relies on its leaves for photosynthesis and natural pruning during the young stage, which requires relatively high amounts of N (Ren et al., 2023). Consequently, plants obtain nutrients more efficiently through their absorptive roots. In the mature forest, Chinese fir was in the flowering and fruiting stage, which required substantial amounts of P to synthesize rRNA to ensure efficient photosynthesis in plants (Li et al., 2010). In the over-mature forest, the Chinese fir plantation was in a state of senescence, which resulted in the death of numerous absorptive roots, thus resorbing and transferring N to the transportive roots (Yuan and Chen, 2012). While stand development changed the betweenness centrality of trait networks, its impact on the connectivity proportion among multiple traits was minimal. Some past studies have shown that plants can maintain high nutrient levels by increasing nutrient conservation in aboveground organs and nutrient uptake from underground organs, suggesting a coordinated relationship between nutrient acquisition by roots and nutrient resorption in leaves (Kou et al., 2017). Although aboveground and underground nutrient resorption capacities of plants showed obvious divergence at different developmental stages, this did not alter the relationship between aboveground and underground nutrient uptake.

### 4.3 Stand development and stand structure co-regulates nutrient resorption efficiency

Our results indicated that the developmental stage not only had a positive effect on stand structure but also exerted a negative effect on RE_AG_ and RE_UG_. Most importantly, there was a positive correlation between the RE_AG_ and RE_UG_, which is consistent with the second hypothesis. RE_AG_ and RE_UG_ showed decreasing trends with stand development, indicating that nutrient accumulation in the aboveground and underground organs of Chinese fir was sufficient to maintain normal growth (Song et al., 2022). Stand structure was also negatively correlated with RE_AG_ and RE_UG_. First of all, this may be due to the positive effect of stand development on stand structure, which indirectly leads to its negative effect on RE_AG_ and RE_UG_; Secondly, although the increase of stand density, relative growth rate and dead twig amount increased RE_AG_ and RE_UG_. However, the crown ratio significantly reduced these factors, and its impact was found to be greater than that of the other factors. The stand density was high in the young and middle forests, resulted in a low crown length and high natural pruning intensity, and the nutrient concentrations of the aboveground and underground organs were generally low; therefore, it was necessary to improve the nutrient resorption capacity (Di et al., 2018). Although the nutrient concentration in the Chinese fir constantly changed with stand development, the nutrient resorption of leaves and roots had a synergistic effect, which implied that plantations improved RE_UG_ through evolutionary nutrient strategies, thereby absorbing more nutrients from the litter to maintain physiological processes (Peri et al., 2006). A previous study suggested that the increase in RE_L_ might be accompanied by a change in the underground nutrient strategy from a “passive” strategy dependent on mycorrhizal fungi to an “active” strategy dependent on the absorption by roots themselves (Zheng et al., 2024). Therefore, stand age and structure play key roles in shaping the relationship between RE_AG_ and RE_UG_.

In general, the effect of developmental stage on RE_UG_ was greater than that of stand structure, which is consistent with the third hypothesis. However, RE_AG_ demonstrated statistically equivalent sensitivity to developmental stage and stand structure. The reason for this result may be that stand structure has a higher degree of influence on RE_AG_. In this study, stand structure was characterized by four key metrics: stand density, relative growth rate, crown ratio, and dead twig biomass. First, an existing study has confirmed that RGR substantially enhances foliar NRE and PRE (Wu et al., 2020). Second, empirical study has demonstrated a unimodal relationship between foliar nutrient concentrations and stand density, with initial increases followed by subsequent declines (Fang et al., 2017), ultimately influencing RE. In summary, RE_AG_ was not less affected by stand structure than developmental stage. Furthermore, in young and middle forests, limited growth space leads to intensified intraspecific competition, resulting in reduced nutrient accumulation within the plants (Brant and Chen, 2015). Therefore, it is necessary to enhance the RE_AG_ in order to obtain the nutrients required for growth. Stand structure also changes during the stand development process. The photosynthetic carbon fixation ability of the aboveground organs was obviously strengthened, and the nutrient concentration in plants also increased to sustain growth, which reduced the nutrient resorption ability (Hayes et al., 2014). As the main organ for the underground absorption of nutrients, fine roots are transported to aboveground organs, resulting in more vigorous physiological activities (Ren et al., 2023). In this study, the RE_UG_ gradually decreased with stand development. First, the nutrient concentration of fine roots generally increased with increasing stand age, implying that nutrient accumulation in Chinese fir was sufficient to maintain growth. Second, Chinese fir has developed a unique mechanism for internal nutrient redistribution and storage through long-term survival competition (Zhou et al., 2015). All in all, the source of nutrients required for Chinese fir growth may change with stand development.

## 5 Conclusion

In this study, the NRE was found to be generally high in the young forest and decreased with stand development, which may be affected by the demand for N at different developmental stages of Chinese fir. The PRE of leaves and twigs was high in the middle and mature forests and lower in the other stages, which may also be related to the demand for P during the flowering and fruiting stages of Chinese fir. With stand development, the hub traits with the highest betweenness centrality in the network transitioned from NRE_AR_ to PRE_L_ and NRE_TR_. This shift indicated that plants required distinct nutrient acquisition strategies at different development stages to maintain normal growth. Specifically, in young and middle forests, Chinese fir exhibited high N demand, which was met through efficient nutrient acquisition by absorptive roots. In mature forest, Chinese fir was in the flowering and fruiting stage, which depended on substantial amounts of P to synthesize rRNA. In over-mature forests, Chinese fir was in a state of senescence, which caused death of numerous absorptive roots, thus resorbing and transferring N to transportive roots. Moreover, there was a synergistic effect seen between the aboveground and underground organs in regard to nutrient resorption, which was caused by the fine roots absorbing nutrients for the growth of twigs and leaves. Finally, the process of stand development and stand structure had the same effect on RE_AG_, whereas RE_UG_ was mainly regulated by the developmental stage because of the root nutrient absorption strategy. The stand structure had a positive effect on RE_AG_ and RE_UG_ through the negative effect of crown ratio and the positive effects of stand density, relative growth rate, and dead twig biomass. In conclusion, when constructing Chinese fir plantations in nutrient-poor environments, we can adopt targeted measures to improve nutrient utilization efficiency and enhance stand productivity according to the requirements of developmental stage. Specific recommendations are as follows: In the young forest, we can either increase the initial planting density or cut alive twigs of the understory canopy in advance to enhance nutrient resorption. In the middle and over-mature forests, which are more limited by nitrogen, and it is suggested to add more nitrogen fertilizer in the soil. In the mature forest, the addition of phosphorus fertilizer is more beneficial for promoting nutrient cycling in Chinese fir plantations.

## Data Availability

The raw data supporting the conclusions of this article will be made available by the authors, without undue reservation.
